# Incidental detection of appendiceal mucocele during colonoscopy: A case report

**DOI:** 10.1016/j.ijscr.2025.111488

**Published:** 2025-06-09

**Authors:** Ahmed Alakeel, Alanoud Mubarah, Mohammed Alshehri, Feras Alsannaa

**Affiliations:** a General Surgery Senior Registrar, Surgery Department, Prince Sultan Military Medical City, Riyadh, Saudi Arabia; b General Surgery Senior Registrar, Ministry of Health, Riyadh First Health Cluster, Riyadh, Saudi Arabia; c General Surgery Senior Registrar, Ministry of Health, Riyadh Third Health Cluster, Riyadh, Saudi Arabia; d Trauma and Acute Care Surgery Consultant, Surgery Department, Prince Sultan Military Medical City, Riyadh, Saudi Arabia

**Keywords:** Appendicular mucocele, Pseudomyxoma peritonei, Laparoscopy, Case report

## Abstract

**Introduction and importance:**

Appendiceal mucocele is a rare condition characterized by the accumulation of mucus within the appendix. It often presents with vague and non-specific symptoms, making its diagnosis and management challenging. The serious complication of this condition is pseudomyxoma peritonei, which can occur if the mucocele ruptures and disseminates mucus throughout the abdominal cavity.

**Case presentation:**

In this case, a 37-year-old woman presented with nonspecific abdominal pain, leading to the incidental discovery of an appendiceal mucocele during a diagnostic workup. The patient underwent a successful laparoscopic appendectomy combined with partial cecectomy, and her post-operative recovery was uneventful.

**Clinical discussion:**

The surgical management of appendiceal mucocele is still controversial. The aim is to avoid rupture, which can cause peritoneal dissemination and the development of pseudomyxoma peritonei. Depending on the extent of the mucocele and the presence of malignant features, treatment can range from simple appendectomy to more extensive procedures such as right hemicolectomy (Gopalan et al., 2024 [4]). Laparoscopic surgery, though minimally invasive, requires meticulous handling to avoid spillage of the mucocele's contents.

**Conclusion:**

Given the potential complications, appendiceal mucocele should be included in the differential diagnosis once more common causes of abdominal pain are excluded. Early and accurate preoperative diagnosis is critical for guiding surgical management, as it helps prevent rupture and ensures the selection of the most appropriate surgical approach, minimizing the risk of further complications.

## Highlights

• Appendiceal mucocele is a rare entity with non-specific symptoms. So, diagnosis can be difficult.
• Appendiceal mucocele may present a challenging clinical situation.
• Surgical resection remains the treatment of choice and plays a potentially curative role.

## 1 Introduction and importance

Appendiceal mucocele is a rare pathological condition in which the appendix gradually enlarges due to an accumulation of mucus in its lumen. It accounts for only 0.2–0.3 % of appendectomy cases a spectrum of benign and malignant diseases [1]. It may be caused by several factors, including luminal obstruction, carcinoid tumors, or even endometriosis. A gradual accumulation of mucus can lead to significant enlargement of the appendix, with potential complications in the form of rupture and pseudomyxoma peritonei [2].

Clinically, appendiceal mucoceles are asymptomatic and often found incidentally by imaging, endoscopy, or surgery. They sometimes mimic acute appendicitis or nonspecific abdominal discomfort, right lower quadrant pain, or gastrointestinal bleeding. Thus, a preoperative diagnosis can be achieved to guide the surgical approach [3]. In this regard, ultrasonography, computed tomography (CT), and colonoscopy will all prove invaluable in confirming and characterizing a mucocele.

The surgical management of appendiceal mucocele is still controversial. The aim is to avoid rupture, which can cause peritoneal dissemination and the development of pseudomyxoma peritonei. Depending on the extent of the mucocele and the presence of malignant features, treatment can range from simple appendectomy to more extensive procedures such as right hemicolectomy [4]. Laparoscopic surgery, though minimally invasive, requires meticulous handling to avoid spillage of the mucocele's contents.

This case report presents the diagnostic and therapeutic course of a 37-year-old woman who initially presented with nonspecific abdominal and anal symptoms and subsequently underwent a workup for suspicion of inflammatory bowel disease leading to the incidental diagnosis of an appendiceal mucocele. This case has been reported in line with the SCARE checklist.

## 2 Case presentation

A 37-year-old normotensive, non-diabetic female from a middle socio-economic class presented with intermittent vague abdominal pain, anal pain with bloody discharge, and swelling around the anus. The patient denied nausea, vomiting, loss of appetite, or weight loss. The review of other systems was unremarkable.

**Fig. 1:**
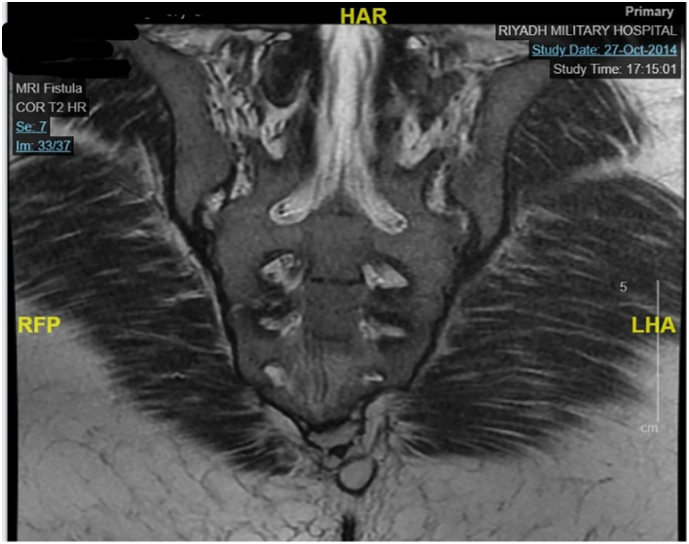
Pelvic magnetic resonance imaging (MRI) showing a pilonidal sinus.

On clinical examination, the patient was found to be vitally stable, well-appearing, oriented, and cooperative, with average build and nutrition. There were no signs of jaundice, bony tenderness, lymphadenopathy, or organomegaly. The abdomen was soft and non-tender without any peritoneal signs. A per rectal (PR) examination revealed a sinus opening at the 7 o'clock position, suggestive of a pilonidal sinus, and external hemorrhoids at the 3 o'clock position, as seen in Fig. 1.

**Fig. 2:**
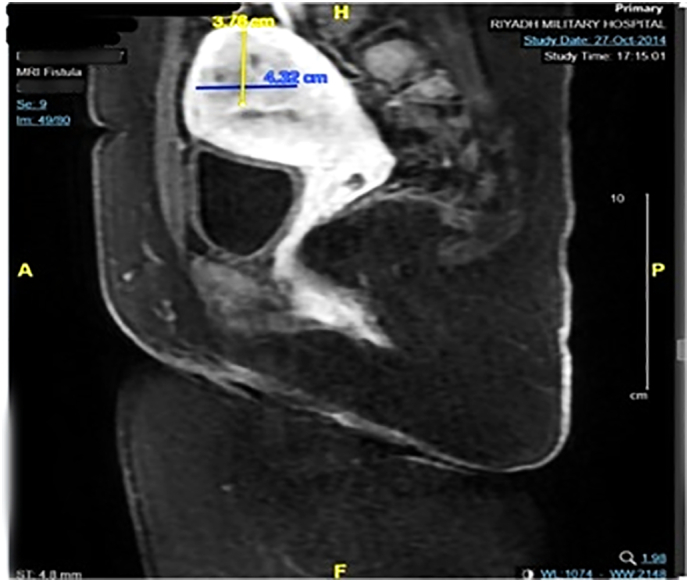
Pelvic MRI showing a uterine fibroid.

Lab tests were within normal limits on the initial presentation. Magnetic resonance imaging (MRI) was done to assess the fistula and determine if there was communication with the sinus. Fig. 2 depicts the MRI findings that present as an oval cystic lesion measuring 3.4 × 14 × 8 mm, compatible with a pilonidal sinus without fistulous communication. In addition, the MRI incidentally detected an ill-defined mass within the posterior superior uterine wall, measuring 3.2 × 4.3 cm with hemorrhagic areas, suggestive of endometriosis. A smaller, well-defined lesion in the anterior uterine wall, measuring 14 mm in diameter, was a fibroid. The appendix was not visualized due to the MRI's pelvic confinement.

**Fig. 3:**
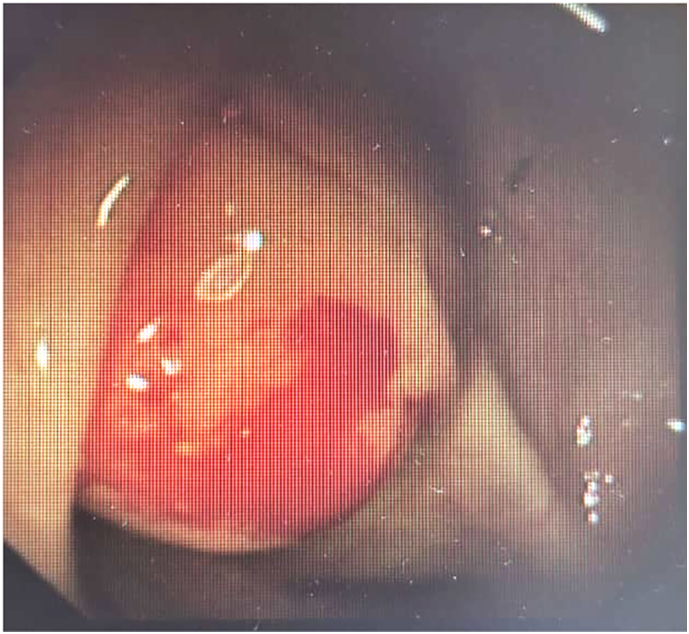
Colonoscopy showing appendicular mass.

After this, a colonoscopy was performed to rule out inflammatory bowel disease. During this, an appendicular mass was found, as in Fig. 3. The biopsy was performed, and the histopathology report showed benign colonic mucosa with mild chronic inflammation and focal crypt distortion, no granulomas, dysplasias, or neoplastic changes, and no appendix tissue.

**Fig. 4:**
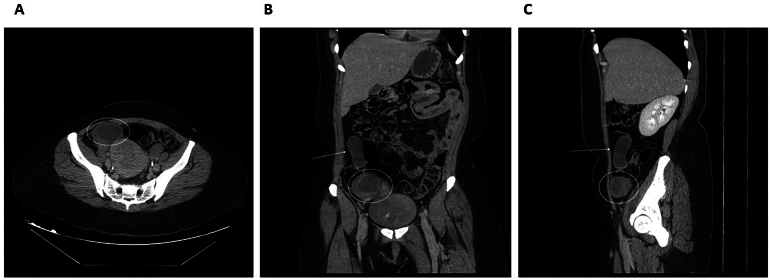
CT scan showing appendicular mass and elongated cystic lesion. A. Axial view, B. Coronal view, C. Sagittal view.

A contrast-enhanced computed tomography (CECT) scan work-up revealed an elongated cystic lesion in the appendix measuring 6.4 cm with a thin enhancing wall but no mural nodules, septation, or soft tissue component; this was consistent with an appendiceal mucocele, as demonstrated in Fig. 4*.* There was no regional lymphadenopathy or peritoneal disease.

All tumor markers, such as CEA (Carcinoembryonic Antigen), CA 19-9 (Carbohydrate Antigen 19-9), and AFP (Alpha-Fetoprotein), were within normal limits. She then had a laparoscopic exploration with partial cecectomy to ensure negative margins. The same surgery addressed a drained perianal abscess. Her postoperative course was uneventful, and she was discharged in good condition on the third postoperative day.

Histopathological examination confirmed the diagnosis of mucinous cystadenoma with mucocele. High-grade dysplasia was not seen, and any invasive features were also lacking. The resection margins were neoplasia free. There was no recurrence in one year of follow-up. Routine clinical evaluation remained unremarkable, with normal CT imaging, and tumor markers remained normal.

## 3 Clinical discussion

Appendiceal mucocele is an extremely rare, slow-growing disease wherein the appendix distends and becomes distended with mucus because of obstruction of the lumen and sometimes for some causes such as endometriosis or carcinoid tumors [5,6]. The histological subtypes of appendiceal mucocele include four: simple mucocele (retention cyst) without hyperplasia or atypia, hyperplastic mucocele with mucosal proliferation, mucinous cystadenoma (the most common type) with dysplasia, and mucinous cystadenocarcinoma, which is marked by high-grade dysplasia and stromal invasion [7].

Appendiceal mucocele is usually incidental in its often-asymptomatic course. It presents symptoms similar to acute appendicitis, including right lower quadrant pain, nausea, or vomiting [4]. It may be rarely associated with gastrointestinal bleeding and intussusception. In this case, the condition was misdiagnosed at first with abdominal symptoms, and the incidental appendiceal mass was discovered during an inflammatory bowel disease checkup [8,9].

Proper preoperative diagnosis prevents appropriate surgical intervention; thus, it would not occur in problems like peritoneal dissemination and intraoperative or postoperative morbidity [9]. Most probably, the first diagnostic modality applied for a case with acute abdominal pain is ultrasonography. In that condition, this mucocele of the appendix appears mostly with an appendicular diameter larger than 15 mm. In ultrasonic studies, the “onion skin” sign is used specifically in the mucocele of the appendix. It is more diagnostic as it has an appendicular lumen diameter larger than 1.3 cm, cystic dilation, and wall calcification. All these features are very suggestive of mucocele. Colonoscopy may easily identify the classic “volcano sign,” wherein the yellow mucus exuding from the appendiceal orifice supports the diagnosis [10].

The choice between open and laparoscopic surgery is contentious. The main aim of surgical intervention is to prevent the rupture of the mucocele, which would spread its contents and significantly increase the risk of developing pseudomyxoma peritonei [5,6]. The advantage of doing this surgery laparoscopically is that it is minimally invasive and, at the same time, allows proper abdominal cavity assessment. Extreme caution has to be exercised while opening the mucocele content to avoid spillage into the abdominal cavity. Sometimes, a simple appendectomy will have to be done, while a much more extensive right hemicolectomy, dependent upon perforation being there and involvement of appendiceal base and lymph node station in the mesentery. If the tumor markers are elevated or the mucocele has a malignant appearance, then a right hemicolectomy is justified. If there is an intraoperative spill, HIPEC (Hyperthermic Intraperitoneal Chemotherapy) is frequently performed to treat the developing possibility of pseudomyxoma peritonei [11].

The patient's mucocele was intact with no sign of perforation or spill, and the resection margins were neoplasia-free. No involvement of the lymph nodes means that a simple appendectomy would be an appropriate intervention for this patient. Given that the pathology is benign, no long-term follow-up was indicated for this patient based on the algorithm. However, studies from retrospective reviews of cases of appendiceal mucocele reveal that even in benign mucoceles, follow-up now and then might be useful to watch out for recurrence or development of pseudomyxoma peritonei. In benign mucoceles, the outcomes after surgery are very good, with survival at 5 years between 91 and 100 % [12]. In malignant mucoceles, survival is significantly reduced, and the range goes from 18.7 % to 55 % [1,12]. Good immediate results can be seen postoperatively, although follow-up for a long time is generally recommended since late recurrence or even the development of pseudomyxoma peritonei may occur up to 21 months post-surgery [3].

Follow-up was recommended for all patients aged 5 to 10 with physical examination, tumor markers such as CEA and CA 19-9, and annual imaging. This allows for early detection and management of complications or synchronous malignancies in delayed recurrences [13,14].

## 4 Conclusion

This case report highlights the rare presentation of an appendiceal mucocele, incidentally diagnosed during a workup for inflammatory bowel disease. The patient's presentation with vague abdominal and anal symptoms initially masked the underlying appendiceal pathology. Imaging studies, particularly MRI and CT scans, were crucial in identifying the lesion and ruling out other conditions. Surgical management via laparoscopic partial cecectomy successfully addressed the mucocele, with histopathology confirming a benign mucinous cystadenoma. No recurrence or complications were observed during one year of follow-up, emphasizing that early diagnosis and appropriate surgical intervention can lead to favorable outcomes in benign appendiceal mucoceles. This underscores the importance of careful surgical handling to prevent rupture and subsequent complications such as pseudomyxoma peritonei. Hence, this case mandates continued vigilance in managing appendiceal mucoceles, balancing minimally invasive approaches with carefully considering potential risks.

## Consent

Written informed consent was obtained from the patient for publication of this case report and accompanying images. A copy of the written consent is available for review by the Editor-in-Chief of this journal on request.

## Ethical approval

In our institute, ethical approval is exempted as it is acquired from patient's consent.

## Sources of funding

This research did not receive any specific grant from funding agencies in the public, commercial, or not-for-profit sectors.

## Research registration

We do not need to register this work.

## Declaration of competing interest

All authors have no conflict of interest.
